# Optical Coherence Tomography Findings in the Retinas of SOD1 Knockout Mice

**DOI:** 10.1167/tvst.9.4.15

**Published:** 2020-03-18

**Authors:** Marco Augustin, Danielle J. Harper, Conrad W. Merkle, Martin Glösmann, Christoph K. Hitzenberger, Bernhard Baumann

**Affiliations:** 1 Center for Medical Physics and Biomedical Engineering, Medical University of Vienna, Vienna, Austria; 2 VetCore Facility for Research, Imaging Unit, University of Veterinary Medicine Vienna, Vienna, Austria

**Keywords:** retinal pigment epithelium, imaging/image analysis, nonclinical, OCT/OCT angiography

## Abstract

**Purpose:**

The retinal phenotype of popular mouse models mimicking ophthalmic diseases, such as the superoxide dismutase 1 (SOD1) knockout (KO) mouse model, has mainly been assessed by ex vivo histology and in vivo fundus photography. We used multifunctional optical coherence tomography (OCT) to characterize the retinas of SOD1 KO mice in vivo.

**Methods:**

The custom-made ophthalmoscope featured a combination of conventional OCT, polarization-sensitive OCT, and OCT angiography. Seven SOD1 KO mice and nine age-matched controls were imaged between 6 and 17 months of age. A postprocessing framework was used to analyze total and outer retinal thickness changes. Drusenlike lesions were segmented, and their sizes and the number of lesions were assessed quantitatively. Their appearance in the conventional reflectivity images, as well as in the corresponding polarization-sensitive images, was characterized qualitatively.

**Results:**

Drusenlike lesions increased in size and number with age for SOD1 KO mice. Exploiting the multiple contrast channels, the appearance of the lesions was found to resemble pseudodrusen observed in eyes of patients suffering from dry age-related macular degeneration. The total and outer retinal thicknesses were lower on average after 11 months and 7 months in SOD1 KO mice compared with age-matched controls. Neovascularizations were found in one out of seven KO animals.

**Conclusions:**

OCT imaging proved beneficial for a detailed in vivo characterization of the pathological changes in SOD1 KO mice.

**Translational Relevance:**

Phenotyping of animal models using modern imaging concepts can be conducted with more precision and might also ease the translation of conclusions between clinical and preclinical research.

## Introduction

Age-related macular degeneration (AMD) is the leading cause of irreversible moderate or severe vision loss in the aged population (>50 years).^1^ Animal models play an important role in the basic understanding of the pathophysiology of AMD and enable the preclinical testing of the safety and efficacy of novel therapeutics.^2^ Genetically engineered murine models are a popular choice for such studies, in which genes that are hypothesized to play a key role in AMD pathogenesis are modified and their phenotypic manifestations are examined.^2^ Genetically engineered mouse models for dry AMD are targeting genes, which are, among others, associated with juvenile macular dystrophies, inflammatory processes, or oxidative stress.^2^ Superoxide dismutase (SOD) is an antioxidant system protecting the retina from oxidative damage. Among the three SOD isoenzymes, Cu, Zn-SOD (SOD1), Mn-SOD (SOD2), and extracellular SOD (SOD3), SOD1 has the highest activity in the retina. Hence SOD1 knockout (KO) mice (SOD1^−/−^) were hypothesized to express age-related retinal pathology. Drusen, choroidal neovascularization and retinal pigment epithelium dysfunction were described for SOD1 KO (Sod1^tm1Leb^) mice using fundus photography and histology by Imamura et al. 2006.^3^

Preclinical imaging of the murine retina is often performed ex vivo using histology or in vivo using fundus photography. High-resolution, three-dimensional, multimodal imaging of the murine retina is still rare, despite recent developments having demonstrated the potential of novel imaging tools for preclinical retinal imaging.^4^^–^^9^ Moreover, clinical multimodal retinal imaging has been shown to improve the visualization and characterization of hallmarks of AMD, such as neovascularizations^10^ or drusen.^11^^,^^12^ A phenotypic characterization of mouse models using modern imaging modalities, such as optical coherence tomography (OCT), is desirable as such methods enable noninvasive, longitudinal imaging of the same eye, and can assess the functional status of the murine eye with resolution in the micrometer range. Furthermore, OCT imaging of the murine retina eases the translation of preclinical findings to their clinical analog by analyzing the structures with the same imaging contrast and resolution.

In this work, we demonstrate high resolution OCT imaging for analyzing the phenotype of SOD1 KO mice. The experimental OCT ophthalmoscope combines conventional OCT to assess the retinal morphology with high resolution,^13^ OCT angiography (OCTA)^14^ to highlight blood vessels, and polarization-sensitive (PS) OCT^15^^,^^16^ for visualizing pigmented structures. Simultaneous use of the multiple functional extensions of OCT, so-called multifunctional OCT, enabled a comprehensive characterization of pathological findings in the SOD1 KO mice, such as drusenlike lesions, neovascularizations, and retinal thickness changes.

## Methods

### Animals and Experimental Timeline

SOD1^−/−^ mice were purchased from The Jackson Laboratory (Bar Harbor, ME, JAX stock #002972 U.S.)^17^ and heterozygous breeding was established using C57BL/6J (B6) (JAX stock #000664) mice for mating. Genotyping was performed according to the protocol provided by The Jackson Laboratory.^18^ The animals were kept under controlled lighting conditions with a 12 hours dark and 12 hours light cycle at the Division of Biomedical Research, Medical University of Vienna, Austria. All mice were anesthetized using inhaled anesthesia (1.5%–2.0% isoflurane with 1.0–1.5 L/min oxygen) during the imaging procedure (10–30 minutes per animal). Tropicamide (5 mg/mL, AGEPHA Pharma s.r.o., Senec, Slovakia) was applied to each eye prior to imaging for pupil dilation, and the cornea was moistened using artificial eye drops (Oculotect, ALCON Pharma GmbH, Freiburg im Breisgau, Germany) to prevent dehydration during anesthesia. Both eyes of each animal were imaged around the optic nerve head (ONH). When lesions were identified in the real-time display during data acquisition, additional scans at locations in the proximity of the ONH were acquired. All experiments were performed under a protocol approved by the ethics committee of the Medical University of Vienna and the Austria Federal Ministry of Science, Research and Economy (GZ BMBWF-66.009/0216-V/3b/2018), and are in accordance with the ARVO Statement for the Use of Animals in Ophthalmic and Vision Research.

**Figure 1.**
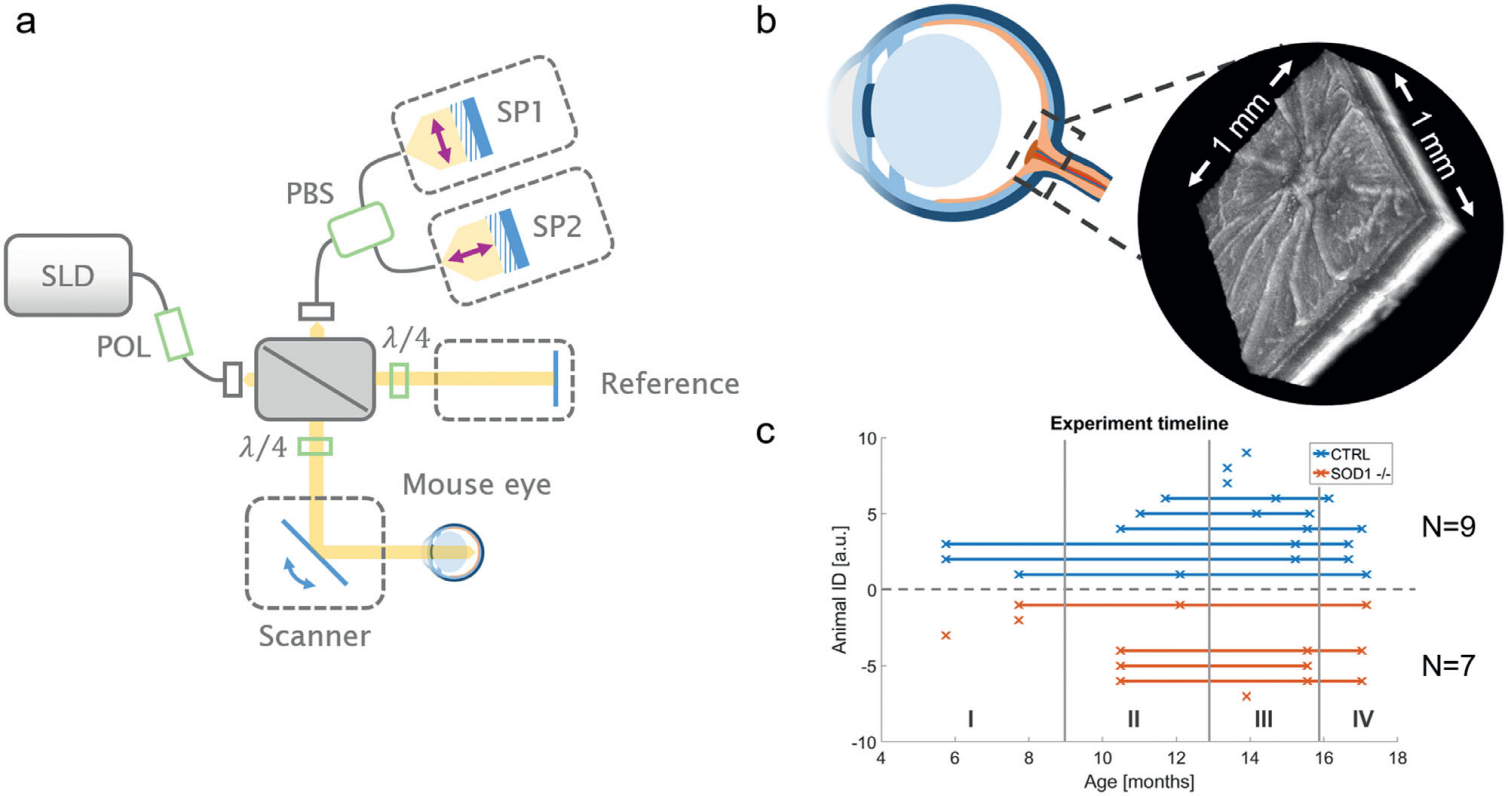
OCT imaging of the mouse eye and experiment timeline. (a) Sketch of the OCT ophthalmoscope comprising a free-space interferometer with polarization optics (green) and two identical spectrometers to acquire the co- and cross-polarization states of the interference signal. (b) A field of view of 1 × 1 mm^2^ around the ONH was imaged. (c) The SOD1 KO mice and age-matched control mice were imaged longitudinally between 6 and 19 months of age. SLD, superluminescent diode; POL, linear polarizer; PBS, polarizing beam splitter; SP1&2, spectrometers; λ/4, quarter wave plate.

The KO group comprised seven homozygous SOD1^−/−^ (n = 7) mice. Six wildtype (n = 6) and three heterozygous littermates (n = 3) were pooled in the control group (CTRL, total n = 9). The two groups were imaged in an age range of 175 days (≈6 months) to 522 days (≈17 months). Longitudinal data with a minimum of two imaging sessions per animal were captured from four KO mice and six CTRL mice. Three KO and three CTRL mice had only one data point. A detailed overview of the experimental timeline is shown in Figure 1c. The longitudinal imaging sessions were clustered in four bins (I-IV) for longitudinal evaluation such that each animal was only represented once per interval. The details of the binned longitudinal data are summarized in the Table, where N is the number of eyes imaged and used for evaluation in the results.

**Table.** Clustering of Longitudinal Image Data into Four Intervals Ranging from 175 Days (≈6 months) to 522 Days (≈17 months) Postnatally

Bin	Age (days)	Mean Age (months)	N (n) SOD1 KO	N (n) CTRL
I	(175–235)	7	6 (3)	6 (3)
II	(319–368)	11	7 (4)	6 (4)
III	(407–475)	15	8 (4)	16 (9)
IV	(491–522)	17	6 (3)	10 (5)

N, number of eyes; n = number of animals.

### Multifunctional OCT Imaging of the Murine Retina

A custom-made PS OCT ophthalmoscope featuring a superluminescent diode (*λ_c_* = 840 nm; ∆*λ* = 100 nm) and two identical spectrometers was utilized. The apparatus is tailored for imaging the rodent retina and was initially described by Fialová et al.^19^ In contrast to conventional OCT systems, the light illuminating the rodent eye has a defined (namely circular) polarization state, and the backscattered light is split by a polarizing beam splitter into the co- and cross-polarization state, after interfering with the light from the reference arm. By analyzing the polarization state of the backscattered light, additional information about the tissue microstructure can be assessed,^14^ for example, pigmented tissue can be visualized by the degree of polarization uniformity (DOPU) parameter.^20^ DOPU values range between 1 (polarization-preserving tissue) and 0 (polarization scrambling). In this work, the DOPU parameter was used to analyze drusenlike lesions and to investigate associated changes in the retinal pigment epithelium (RPE). A simple sketch of the system is illustrated in Figure 1a, where the polarization optics (depicted in green) and the two spectrometers (SP1 and SP2) capturing the co- and cross-polarization state are highlighted as the major modifications compared with conventional OCT systems. The prototype offers an axial resolution of 3.8 µm in retinal tissue and a field of view of 1 × 1 mm^2^ on the mouse fundus was imaged (Fig. 1b). In addition to the conventional OCT reflectivity and the PS contrast, OCTA was simultaneously performed by analyzing the complex OCT signal of repeated B-scans. A detailed description of the multifunctional imaging contrast, the scanning protocol, and the postprocessing framework can be found elsewhere.^21^ The postprocessing pipeline was extended to include a retinal layer segmentation algorithm that uses both reflectivity and polarization information.^22^ Using this algorithm, five retinal surfaces were segmented, namely the inner limiting membrane (ILM), the inner and outer surfaces of the outer plexiform layer (OPL), and the inner and outer surfaces of the RPE. From this segmentation, the total retina, defined as the region between the ILM and the RPE posterior surface, as well as the outer retina, defined as the region between the OPL posterior surface and the RPE posterior surface, were determined. The thicknesses of these features were evaluated as mean values in a 200-µm wide annulus around the ONH.

### Drusenlike Lesion Analysis

Drusenlike lesions form in the outer retina of SOD1 KO mice^3^ and appear as hyper-reflective spots in OCT B-scan images (see section Results, Drusenlike Lesions). To analyze the presence of these lesions, the reflectivity images were postprocessed to enhance the contrast of the lesions prior to manual segmentation. The following steps were implemented in MATLAB (R2015b) (MathWorks, Natick, MA).

**Figure 2.**
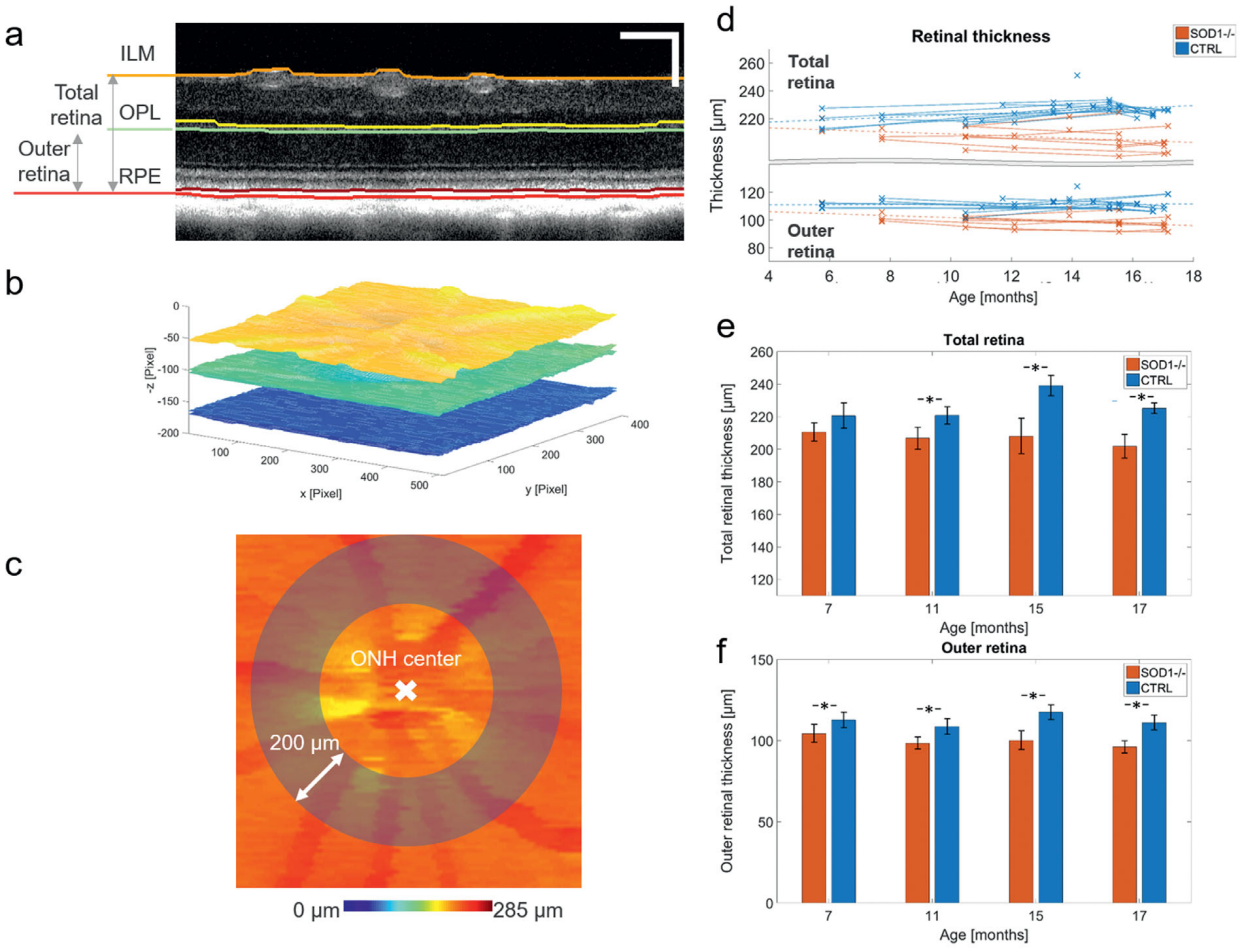
Retinal thickness analysis in SOD1 KO mice and age-matched controls. (a) Five retinal interfaces were segmented. For our analysis, the total retina and the outer retina were defined to range from ILM to the posterior RPE surface and from OPL to posterior RPE surface, respectively. Scale bar = 100 µm. (b) Layer segmentation was performed for the whole volume. (c) A 200-µm wide annulus was used to assess the average layer thickness for each volume. (d) Individual total and outer retinal thickness measurements for the CTRL and KO groups. Linear regressions for the KO and CTRL group are plotted as dashed lines. (e-f) Longitudinal image data were clustered by age into four bins. Total and outer retinal thickness was significantly thinner for the KO group in all age groups (*P* *<* 0.05), except for the total retinal thickness in the first bin (*P* = 0.06). Significant changes between groups are marked with *. Bar chart shows mean values where the error bars indicate ± standard deviation.

1. Reflectivity images (in log scale) were flattened and segmented as described in previous literature^21^^,^^22^ and only the outer retinal slab, defined between the OPL and the posterior surface of the RPE (Fig. 2a), was used for the subsequent steps.
2. The background of each en face (x-y) plane was estimated using a circular average filter with a 20-pixel radius and was subtracted from the original signal.
3. The background-subtracted images were smoothed in the en face plane using a median filter with a 5 pixels × 5 pixels kernel.
4. The resulting image stacks were loaded into Fiji^23^ and the Segmentation Editor plugin was used to segment the hyper-reflective areas in the en face plane 10 µm anterior to the RPE.
5. After the segmentation in the en face plane, the B-scans containing lesions and the neighboring frames were manually inspected and falsely segmented lesions were removed.
6. The number of lesion, and the total lesion area was evaluated in an annulus (inner diameter = 500 µm; outer diameter = 900 µm) centered at the ONH (Fig. 2c). In addition, the individual lesions area was evaluated for all eyes over time.

### Statistical Analysis

The total and outer retinal thickness in addition to the number of lesions and lesion area were compared between the KO and the CTRL groups using the nonparametric Wilcoxon rank-sum test in R version 3.5.2. (R Foundation for Statistical Computing, Vienna, Austria). *P* values <0.05 were considered statistically significant. All values are given as mean values (± standard deviation) unless otherwise stated.

## Results

### Retinal Thickness Changes

Total and outer retinal thickness were reduced for SOD1 KO mice compared with the CTRL group over the whole experimental timeline. An illustration of the retinal thickness analysis is depicted in Figures 2a–c. The total and outer retinal thickness for the individual eyes of each group are shown in the top and bottom panels of Figure 2d, respectively. The total retina of the KO group was significantly thinner than the CTRL group at 11, 15, and 17 months of age (all *P* *<* 0.01), but not at 7 months after birth (*P* = 0.06). The outer retina was significantly thinner in the KO group at all time points (all *P* *<* 0.05). The total retinal thickness for the KO group was 210 (± 6) µm and 202 (± 7) µm for the measurements of the first and fourth bin, respectively (linear regression model: –0.8 µm/month, *P* = 0.07, R² = 0.12). For comparison, the total retinal thickness for the CTRL group was 219 (± 6) µm and 224 (± 2) µm for the measurements of the first and fourth bin (linear regression model: + 0.8 µm/month, *P* < 0.01, R² = 0.20). The thickness of the outer retina for the KO group similarly decreased from 104 (± 6) µm to 96 (± 4) µm between the first and the fourth bin (linear regression model: –0.7 µm/month, *P* < 0.01, R² = 0.27). The thickness of the outer retina for the CTRL group stayed constant and was measured as 112 (± 3) µm and 111 (± 5) µm for the first and the fourth bin (linear regression model: + 0.0 µm/month, *P* = 0.84, R² = 0.00).

### Drusenlike Lesions

**Figure 3.**
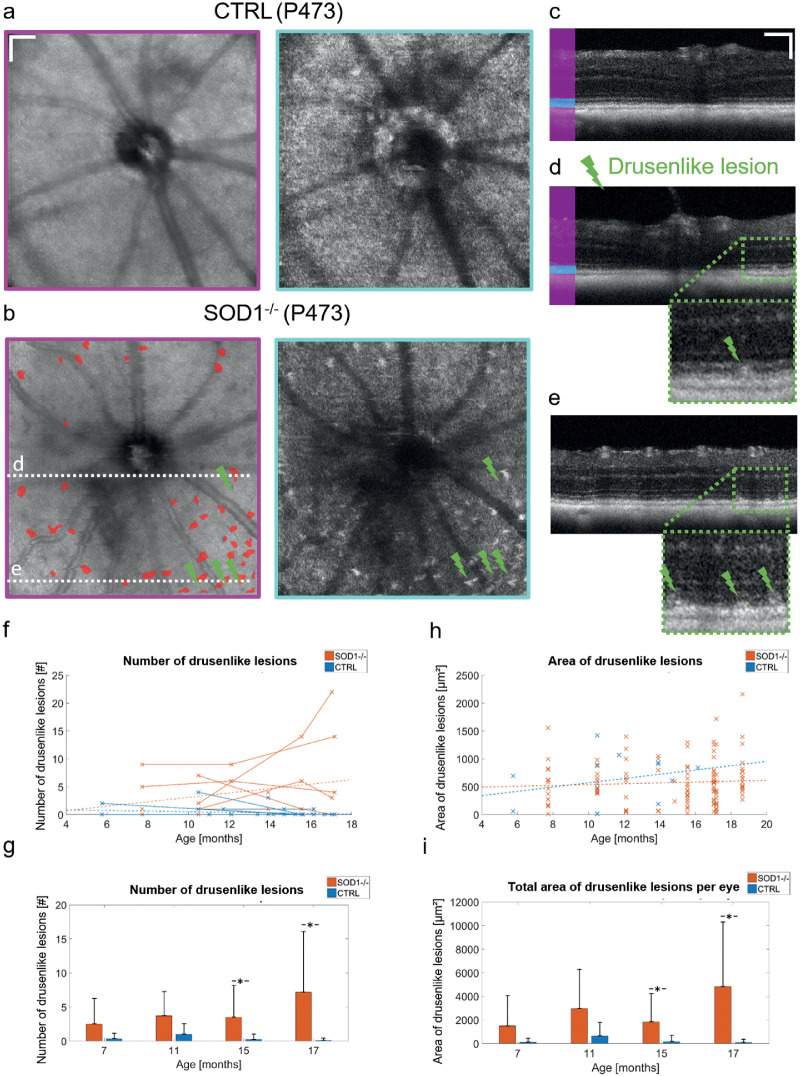
Appearance of drusenlike lesions in OCT images of SOD1 KO retinas. (a) Average en face projection for the whole posterior eye and a 10-µm slab anterior to the RPE for a CTRL mouse and (b) an SOD1 KO mouse. Hyper-reflective lesions are visible in the KO retina. Segmentation of the lesions are shown in the total retina projection. (c) Tomogram indicating the slab for the projection used in (a). (d, e) Two tomograms with drusenlike lesions. The respective positions of the tomograms are indicated in (b). Drusenlike lesions are indicated by the green flashes. Zoom-ins at individual lesions are shown beneath. (f) Individual lesion counts for all eyes examined in the study. Linear regressions for the KO and CTRL group are plotted as dashed lines. (g) Binned longitudinal data for the number of drusenlike lesions. (h) Development of the area of individual drusenlike lesions over time. Linear regressions for the KO and CTRL group are plotted as dashed lines. (i) The total lesion area per eye was evaluated in the four time intervals. Scale bars = 100 µm. Bar charts show mean values where the error bars indicate the standard deviation. *p < 0.05.

Hyper-reflective lesions were found in the outer retina at the photoreceptor level using OCT reflectivity contrast. Figure 3 shows an average en face projection for the whole posterior eye (magenta) and for a 10-µm slab anterior to the RPE (cyan) in a control mouse (Fig. 3a) and a mutant mouse (Fig. 3b). Isolated bright spots in the depth projection image of the photoreceptor slab are visible in the mutant eye. Two tomograms for the mutant eye are shown in Figures 3d, 3e, with a zoom-in at the location of the drusenlike lesions. The lesions were segmented for all mice and the number of lesions, as well as the total lesion area at the photoreceptor level, was evaluated in the 200-µm wide annulus around the ONH. Hyper-reflective lesions were observed in 10 out of 14 eyes in the KO group (71.4 %) and in 5 out of 18 eyes in the CTRL group (27.8 %). The temporal evolution of individual lesion counts per eye for all animals is shown in Figure 3f. Linear regression analysis showed no significant dependence of the number of lesions on age (CTRL: *P* = 0.19, SOD1 KO: *P* = 0.16).

The binned data for the number of lesions are shown in Figure 3g. The area of the individual lesions showed an increase over time for both groups (Fig. 3h). Linear regression analysis showed no significant dependence of the individual lesion area on age (CTRL: *P* = 0.37, SOD1 KO: *P* = 0.44). The average lesion area per eye is shown in Figure 3i. The number of lesions and the total lesion area were significantly higher for the KO group for the two measurements at older ages (*P* *<* 0.05).

### OCTA Revealing Neovascularization

**Figure 4.**
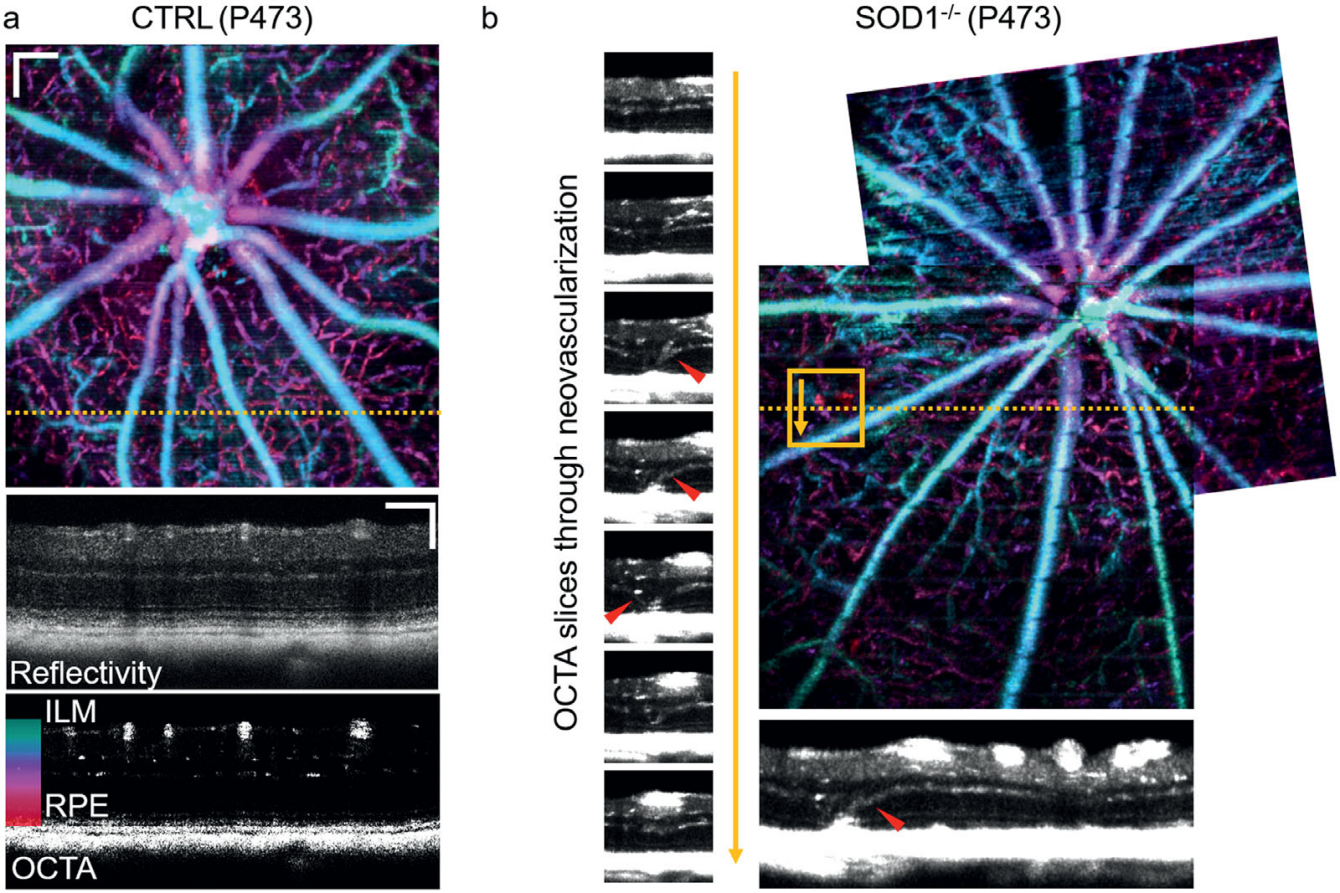
Neovascularization in SOD1 KO mice. (a) Depth encoded color OCTA fundus projection image between the ILM and the RPE in a control mouse. Reflectivity and OCTA tomograms are shown for the location indicated in the en face projection. (b) Depth encoded color OCTA fundus projection image stitched from two scans at neighboring locations in an SOD1 KO mouse. OCTA sections through the neovascularization (indicated in the orange box) revealed the presence of abnormal blood flow in the outer retina indicative of retinal/choroidal neovascularization. These abnormal vessels connecting the inner and outer retina are indicated with red arrowheads.

OCTA revealed the morphology of blood vessel networks in the murine retinas. The organization of the retinal blood vessel network in a control mouse can be observed in a depth-color coded OCTA projection image in Figure 4a. Corresponding reflectivity and OCTA tomograms reveal regular layer morphology and microvascular beds localized exclusively in the inner retina. In one mutant retina, neovascularizations were found in the proximity of the ONH. Two stitched OCTA projections are shown in  Figure 4b, where the neovascularization can be identified by red color coding, indicating the presence of abnormal blood flow in the outer retina. Tomographic projections (maximum of 10 B-scans) from the indicated location show the ascending/descending vessels at the site of neovascularization in greater detail.

### Appearance of Drusenlike Lesions in PS-OCT

**Figure 5.**
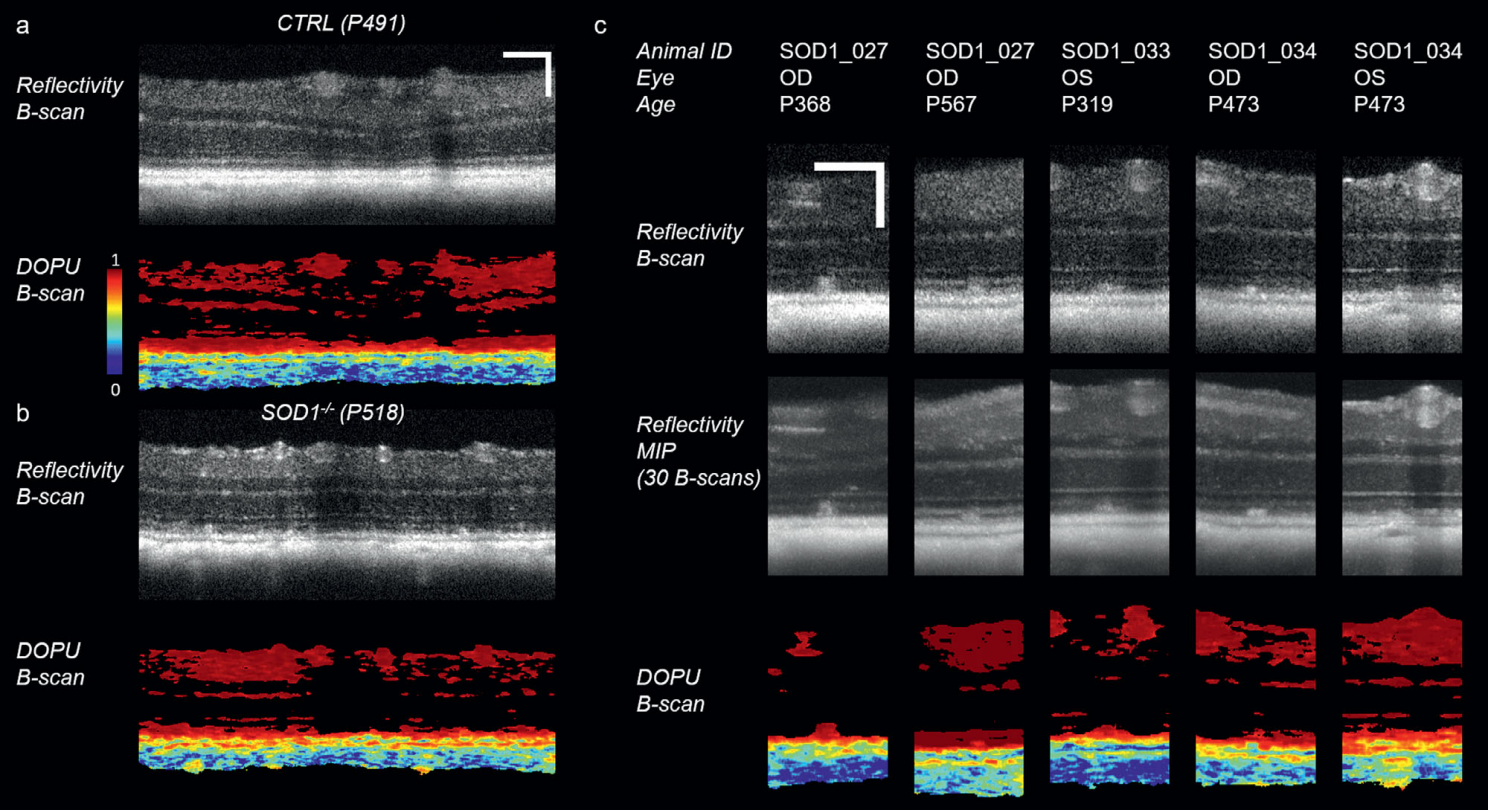
Appearance of drusenlike lesions in SOD1 KO mice in PS-OCT images. (a) Reflectivity tomogram and corresponding DOPU tomogram from a control mouse. Polarization preserving tissue exhibits DOPU values close to 1 and is shown in red, whereas depolarizing tissue (namely the RPE and choroid) produces lower DOPU values indicated by colder colors. Pixels with signal intensity lower than the mean signal in the vitreous plus three times the standard deviation of that signal are shown in black. (b) Reflectivity image from an SOD1 KO mouse. Hyper-reflective material can be observed anterior to the RPE and appears as polarization preserving in the corresponding DOPU image. (c) Five examples of drusenlike lesions in SOD1 KO mice.

Drusenlike lesions were qualitatively analyzed with respect to accompanying RPE changes in the DOPU images. Figure 5a shows a reflectivity B-scan of a control mouse and the corresponding DOPU tomogram. A red color indicates polarization-preserving tissue such as the photoreceptors, yellowish to bluish color indicates depolarizing tissue such as the RPE and the choroid, and pixels with low signal intensity are shown in black. In Figure 5b, a reflectivity B-scan and the corresponding DOPU B-scan are shown for a mutant mouse. Hyper-reflective lesions can be observed in the outer retina in the reflectivity B-scan. The DOPU image of the control and the mutant mouse have a largely similar appearance, apart from the hyper-reflective lesions in the mutant retina, which are polarization-preserving. Figure 5c highlights the appearance of different lesions observed in different mice. The upper row shows reflectivity images of a single B-scan location, the middle row shows a maximum intensity projection from a substack of 30 tomograms around the drusenlike lesions, and the bottom row shows the respective DOPU B-scans at the same location as in the top row. Although the lesions appear with brightness similar to the RPE in OCT reflectivity data, the depolarizing property of the RPE enables its distinction as a continuous layer posterior to the lesions in the DOPU images.

## Discussion

Mice deficient of SOD1 are a popular model of dry AMD.^2^^,^^3^^,^^24^ In this study, we used a multifunctional OCT system to characterize phenotypical changes, which develop in ageing SOD1 KO mice. Drusenlike lesions were found in 71.4% of the outer retinas of aged SOD1 mice in the proximity of the ONH (field of view 1 mm × 1 mm). For comparison, Imamura et al.^3^ detected lesions in 86% (31 of 36 eyes) using fundus photography with a larger field-of-view. In our OCT-based study, the drusenlike lesions were found at the level of the photoreceptors without excessive elevation of internal retinal structures, similar to the lesion morphology observed in AMD patients with subretinal drusenoid deposits (pseudodrusen).^11^^,^^12^ Furthermore, the RPE at the location of the drusenlike lesions seemed mostly unaffected by the lesion formation in the OCT reflectivity images (Figs. 3, 5). This observation is further substantiated by the regular appearance of the RPE in the DOPU images. Although PS-OCT has been proven capable of detecting pigmentation changes such as the migration of melanin^25^^–^^27^ or the elevation of the RPE as in the case of soft drusen,^28^^,^^29^ the drusenlike lesions observed in the SOD1 KO mice exhibited polarization characteristics similar to those found in patients with pseudodrusen: a polarization-preserving, hyper-reflective focal deposit overlying the depolarizing RPE layer (Fig. 5).^28^ The appearance of the lesions in the SOD1 KO mice varied from smaller, flat drusenoid structures to cuticular structures reaching the external limiting membrane (Fig. 5). In this study, a direct correlation to histopathology was not performed, and thus is limiting our conclusions on the histopathological equivalent of the subretinal drusenoid deposits found using OCT. Recent reports on various mouse models of AMD, such as the Ccl2-KO,^30^ the Cx3cr1-KO,^31^ and a triple KO model including SOD1,^32^ have demonstrated that the drusenlike lesions are linked to accumulations of subretinal microglia cells.

In this work, a multifunctional OCT imaging approach was used for in vivo “labeling” of different retinal structures, namely blood vessels and pigmented tissue in addition to the conventional, intensity-based contrast revealing the retinal morphology. Of course, in comparison to conventional histology this number of “stains” is very limited. However, using OCT for longitudinal studies enables the assessment of retinal function and morphology repeatedly over time. This not only decreases the number of animals involved in preclinical studies but also permits a higher precision as the variation in one animal is expected to be lower than the variation in one strain. Nevertheless, neither histology nor OCT is free of artefacts. Although the tissue preparation process can cause folding or shrinkage in histology, OCT is known to suffer from artefacts caused by, for example, sample motion or poor signal quality (e.g., in case of cataract).^26^^,^^33^

In 5 out of 18 eyes from the CTRL, we found hyper-reflective lesions. Although spontaneous age-related changes might be one explanation for these findings, another reason could be inherited defects owing to the genetic background of the wildtype CTRL. Additional genotyping of the CTRL targeting such disorders, for example rd8 mutation that is known to be present in some C57BL/6 lines, was not performed, and hence cannot be ruled out.^34^ Furthermore, we observed that the number of lesions and consequently also the total area of the lesions decreased after 11 months. Although the observed reduction may reflect natural changes over time, it is more likely that some lesions are not detected. An explanation might be physiological temporal changes in the eyes over time, but also that some lesions might not be detected in consecutive measurements owing to the slightly different scanning positions or signal fluctuations.

Retinal thickness changes were observed in SOD1 KO mice and quantitatively assessed based on the volumetric OCT reflectivity data. The total retinal thickness was significantly thinner in the KO mice starting at an age of approximately 11 months when compared with the CTRL. However, the outer retinal thickness was significantly thinner already at 7 months. These findings are in good agreement with previous works. ONL thickness changes for younger mice were found not to be significant at an age of 24 weeks,^35^ 10 weeks,^36^ and 4 months.^24^ Significant thinning of the ONL at approximately 7 months was observed by Hashizume et al.^36^ based on histology, and Carver et al.^24^ found a significant thinning of the total retina between 9 and 12 months of age based on OCT. Although a significant difference compared with the CTRL was found, the total average retinal thinning from 210 to 202 µm was rather subtle when compared with other retinal degenerative models such as the *rd10* mouse model.^5^

Contrary to our expectation, the total retinal thickness in the CTRL did not decrease with age. A possible reason for the stable thickness we observed could be related to physiological parameters (anesthesia tolerance, vasodilation, blood pressure), which might affect the blood vessel size and consequently also the total retinal thickness measurement. Furthermore, retinal layer visibilities and thus thickness measurements may be influenced by signal fluctuations (e.g., due to lens opacities).

Neovascularizations were found in one eye of one mutant animal (1 out of 14; 7%). In comparison, using fundus and histological examination, Imamura et al.^3^ reported exudative lesions in 8.3% and choroidal neovascularization in 10% of SOD1 KO mice older than 10 months. OCTA as shown in Figure 4 enables the three-dimensional investigation of neovascularization in the SOD1 retina in vivo. The cross-section in Figure 4b reveals an obvious connection between the retinal and choroidal vascular beds. This retinal-choroidal anastomosis was found in an SOD1^−/−^ animal aged approximately 16 months.

Retinal phenotyping based on OCT also has some limitations when compared with other methods, such as histology or fundus photography. The system's axial resolution is approximately 4 µm. Other studies^3^^,^^24^ report thickening of Bruch's membrane in the SOD1 KO mice. However, as the thickness of Bruch's membrane is below the resolution of our OCT prototype, subtle thickness changes cannot be assessed in our image data. Switching to other wavelength regions and/or to broader bandwidths would improve the resolution and might offer a way to study changes of Bruch's membrane morphology in the future.^37^^–^^40^ Furthermore, the field of view is limited to approximately 1 × 1 mm^2^, which only allows analysis of a fraction of the retina. Hence the number of lesions in this work are reported for a limited region around the ONH for the longitudinal analysis, and potential lesions in the periphery stayed undetected. In contrast, fundus photography, for example, has a larger expected field of view and can perform angiography to visualize leakage when used in combination with an injection of a fluorescent dye. Current OCTA approaches are not able to detect leakage.

Animal models with induced oxidative damage have a reduced lifespan, muscle weakness, and lower weight.^41^^,^^42^ Longitudinal studies are therefore subject to above average animal dropout and are harder to maintain. In our study, two mice died before the age of 8 months without a known cause. The phenotype of this model has a rather slow progression, mice younger than 7 months were reported indistinguishable from wildtype mice.^3^ Because of this and the increased mortality makes the SOD1 KO model suboptimal for longitudinal retinal studies spanning the whole life cycle of 2 years.

## Conclusions

In this study, multifunctional OCT was used to characterize the phenotype of SOD1 KO mice in vivo. Mice lacking an SOD1 antioxidant system are known to express age-related defects in the retina after 6 months of age. Using noninvasive, longitudinal OCT imaging, an increase in drusenlike lesions with age was observed, and the appearance of the lesions was similar to clinically diagnosed pseudodrusen in patients of dry AMD. Furthermore, the development of neovascularizations was observed, and a significant thinning of the retina was measured. The multiple contrast channels of the OCT system utilized worked together to more completely characterize the phenotypes of SOD1 KO mice in vivo. Analyzing the retinas of mice with modern functional and morphologic OCT ophthalmoscopes, as they are also used in clinical studies, may lead to an improved understanding of drug efficacy and safety aspects during the development of novel ophthalmic therapies, and ease their translation.
